# Mechanism of error-free DNA synthesis across N1-methyl-deoxyadenosine by human DNA polymerase-*ι*

**DOI:** 10.1038/srep43904

**Published:** 2017-03-08

**Authors:** Rinku Jain, Jayati Roy Choudhury, Angeliki Buku, Robert E. Johnson, Louise Prakash, Satya Prakash, Aneel K. Aggarwal

**Affiliations:** 1 Department of Pharmacological Sciences, Icahn School of Medicine at Mount Sinai, Box 1677, 1425 Madison Avenue, New York, NY 10029, USA; 2 Department of Biochemistry and Molecular Biology, 301 University Blvd., University of Texas Medical Branch, Galveston, TX 77755-1061, USA.

## Abstract

N1-methyl-deoxyadenosine (1-MeA) is formed by methylation of deoxyadenosine at the N1 atom. 1-MeA presents a block to replicative DNA polymerases due to its inability to participate in Watson-Crick (W-C) base pairing. Here we determine how human DNA polymerase-ι (Polι) promotes error-free replication across 1-MeA. Steady state kinetic analyses indicate that Polι is ~100 fold more efficient in incorporating the correct nucleotide T versus the incorrect nucleotide C opposite 1-MeA. To understand the basis of this selectivity, we determined ternary structures of Polι bound to template 1-MeA and incoming dTTP or dCTP. In both structures, template 1-MeA rotates to the *syn* conformation but pairs differently with dTTP versus dCTP. Thus, whereas dTTP partakes in stable Hoogsteen base pairing with 1-MeA, dCTP fails to gain a “foothold” and is largely disordered. Together, our kinetic and structural studies show how Polι maintains discrimination between correct and incorrect incoming nucleotide opposite 1-MeA in preserving genome integrity.

Alkylating agents are common reactive chemicals in the environment (e.g. tobacco smoke)^1^,^2^,^3^ and in cells (e.g. S-adenosylmethionine) that can modify the structures of biological macromolecules by transferring alkyl carbon groups^4^. DNA bases can be alkylated at the ring nitrogen and extracyclic oxygen to generate a variety of adducts^5^. N1-methyl-deoxyadenosine (1-MeA) is a mutagenic adduct formed by methylation of deoxyadenosine at N1 (Fig. 1). 1-MeA is particularly pernicious because the N1 atom in adenosine is engaged in Watson-Crick (W-C) base pairing with thymine and its modification by a methyl group impairs W-C base pairing and presents a strong block to normal DNA replication.

Cells have evolved a variety of mechanisms to repair alkylated DNA bases^6^,^7^,^8^. This includes the classical multi-step pathways invoking base excision repair (BER), mismatch repair (MMR), and nucleotide excision repair (NER), as well as specific enzymes that can directly dealkylate the bases. Amongst the latter, AlkB in *E. coli*^9^,^10^ and ABH2 in mammals^11^,^12^,^13^ use a mononuclear iron (II) center and cofactors such as 2-ketoglutarate and dioxygen to demethylate the 1-MeA adduct directly^8^,^14^. Accordingly, mouse embryonic fibroblast lines derived from ABH2 null mice are found to be highly defective in the repair of 1-MeA adducts^15^. However, not all 1-MeA are repaired and will be encountered by the replication machinery.

The Y-family of DNA polymerases allow for the continuity of the replication fork by allowing replication through lesions that impede the replicative polymerases^16^. Humans have four Y-family polymerases – Polι, Polη, Polκ, and Rev1 – each with a unique DNA damage bypass and fidelity profile. Amongst these, Polι stands out in that it does not rely on W-C base pairing between the template base and incoming nucleotide for catalysis. Instead, the active site cleft of Polι is much narrower than in other DNA polymerases, favoring Hoogsteen base pairing^17^,^18^. As such, Polι would appear to be well suited to bypass 1-MeA, which has an altered W-C edge but an intact Hoogsteen edge^19^. Indeed, recent genetic studies in human cells show that translesion synthesis (TLS) across 1-MeA is mediated by three pathways, one of which is dependent on Polι^20^.

We show here by steady state kinetic analysis that Polι exhibits an ~100 fold higher catalytic efficiency for insertion of the correct nucleotide T relative to the incorrect C opposite 1-MeA. We also present ternary structures of Polι bound to template 1-MeA and incoming dTTP or dCTP. We show that template 1-MeA adopts the *syn* conformation in both structures, though with significant differences. dTTP and dCTP insert differently opposite template 1-MeA with dTTP participating in Hoogsteen base pairing, while dCTP is largely disordered, consistent with multiple conformations. Together, our kinetic and structural studies show that Polι can not only accommodate lesions such as 1-MeA with impaired W-C edges, but that it can maintain discrimination between correct and incorrect incoming nucleotides opposite the lesion.

## Results

### Kinetic Analysis

We carried out steady state kinetic analyses to determine the catalytic efficiency (*k*_cat_/*K*_m_) and fidelity of Polι for nucleotide insertion opposite 1-MeA (Table 1). Polι inserts T opposite 1-MeA with an ~5-fold higher catalytic efficiency than opposite undamaged A; however, it also inserts incorrect nucleotides opposite 1-MeA more efficiently than opposite undamaged A. For example, whereas no insertion of C was detected opposite undamaged A, Polι inserts C opposite 1-MeA with a *k*_cat_/*K*_m_ of 0.1 min^−1^ μM^−1^. Importantly, although Polι inserts a C opposite 1-MeA, it does so with a 100 fold lower efficiency than correct T. To understand the ability of Polι to discriminate between T and C opposite 1-MeA, we determined the crystal structures of human Polι bound to a template-primer duplex with 1-MeA as the templating base and dTTP or dCTP as the incoming nucleotide.

### Structure Determination

**Figure 1:**
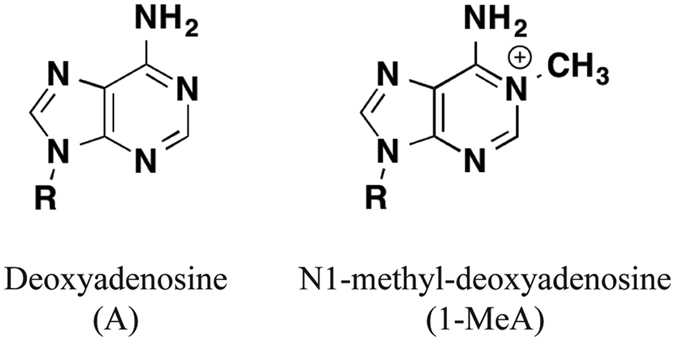
Chemical structure of adensoine (left) and N1-methyl-deoxyadenosine (right).

**Figure 2:**
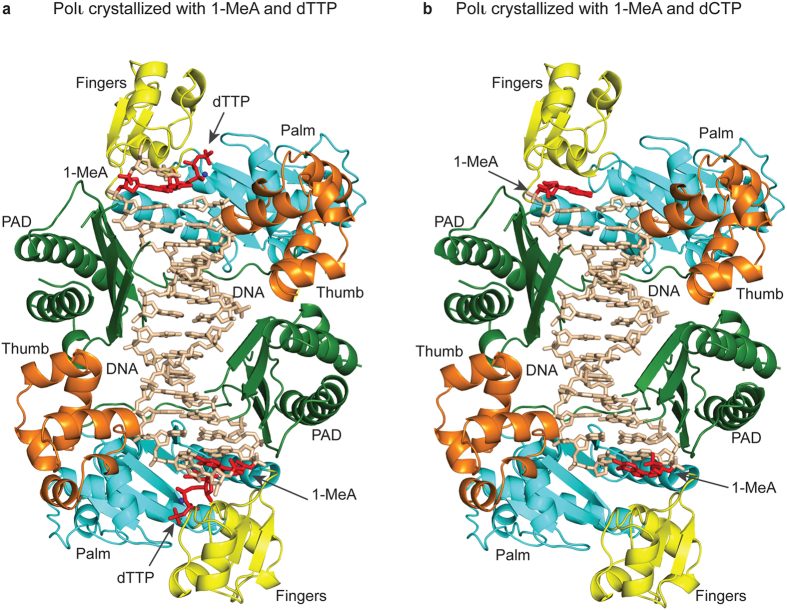
Overall structure of (**a**) Polι_1-MeA.dTTP_ and (**b**) Polι_1-MeA.dCTP_ ternary complexes. In both complexes, a molecule of Polι is bound to each end of the template-primer duplex. Palm, fingers, and thumb domains, and PAD are shown in cyan, yellow, orange, and green respectively. DNA is shown in tan; template 1-MeA is shown in red. For Polι_1-MeA.dTTP_ , incoming dTTP is shown in red and the Mg^2+^ ion at site B is shown as a dark blue sphere.

**Figure 3:**
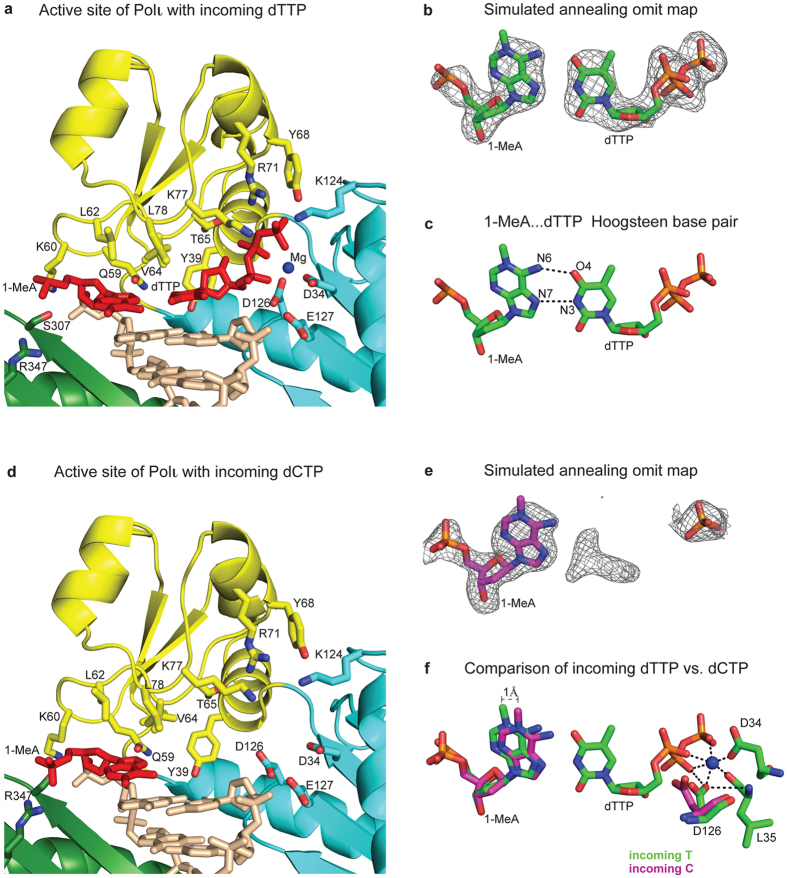
Comparison of active site in the Polι_1-MeA.dTTP_ and Polι_1-MeA.dCTP_ ternary complexes. (Panels a,d) Close-up view of the active site regions in Polι_1-MeA.dTTP_ and Polι_1-MeA.dTCP_ respectively. The catalytic residues (D34, D126, and E127), residues apposed close to the template base (Q59, K60, L62, V64, L78, S307, and R347), and those near the incoming nucleotide (Y39, T65, Y68, R71, and K214) are highlighted and labeled. Template 1-MeA and incoming dTTP are shown in red. (Panels b,e) Simulated annealing F_o_-F_c_ omit maps (contoured at 3σ) around the templating base and the incoming nucleotide in the structures of Polι_1-MeA.dTTP_ (**b**) and Polι_1-MeA.dTCP_ (**e**) respectively. For the incoming dCTP, electron density is very weak with clear density visible only for its γ-phosphate (modeled in orange stick) and poor density visible for the base. (Panel c) 1-MeA.dTTP base pairing in the active site of Polι_1-MeA.dTTP_ ternary complexes. (Panel f) Comparison of 1-MeA…dTTP (green) base pairing in the active site of Polι_1-MeA.dTTP_ with 1-MeA (magenta) in the structure of Polι_1-MeA.dCTP_. Template 1-MeA in the incoming dCTP structure protrudes into the dNTP binding pocket by 1 Å relative to that in the incoming dTTP structure. In Polι_1-MeA.dTTP_ , D126 interacts with the backbone of L35 and with the Mg^2+^ ion at site B. In contrast, in the structure of Polι_1-MeA.dCTP_ , D126 remains in the “binary” like conformation.

To crystallize Polι with 1-MeA, we used an 18-nt template-primer duplex designed to have two identical replicative ends (Fig. 2) and 1-MeA as the templating base (see Methods). Cocrystals with incoming dTTP or dCTP grow under the same conditions (from PEG solutions) and belong to space group P6_5_22, with nearly identical cell dimensions of a = 98.0 Å, b = 98.0 Å, c = 202.5 Å or 202.2 Å (for incoming dTTP and dCTP respectively) and α = β = 90°, γ = 120° (Table 2). The Polι_1-MeA.dTTP_ and Polι_1-MeA.dCTP_ structures were solved by molecular replacement (MR) using the structure of the Polι_A.dTTP_ complex as a search model (PDB ID: 2FLL)^17^ with coordinates of the template A and incoming dTTP omitted. Clear electron density was visible for both the template 1-MeA and the incoming dTTP in initial F_o_-F_c_ and simulated annealing Fo-Fc omit maps for Polι_1-MeA.dTTP_ (Fig. 3). The Polι_1-MeA.dTTP_ ternary complex (R_free_ of 24.9%, R_cryst_ of 20.5%) was refined to 2.6 Å resolution (Table 2) and contains Polι residues 26–350, 356–371, 378–397 and 403–414, DNA nucleotides 3–11, incoming dTTP, 1 Mg^2+^ ion, 1 Cl^−^ ion, and 87 water molecules.

Crystals of Polι_1-MeA.dCTP_ diffracted to 2.0 Å and clear electron density was visible for the templating 1-MeA in the initial F_o_-F_c_ and simulated annealing Fo-Fc omit maps. However, the electron density for the incoming dCTP was not as well-defined as for the dTTP in the Polι_1-MeA.dTTP_ structure, despite the substantially higher resolution (2.0 Å vs. 2.6 Å) of the Polι_1-MeA.dCTP_ structure (Fig. 3e). Strong F_o_-F_c_ density (above 3σ) was visible only for the γ-phosphate of dCTP and partial electron density was observed for what would be considered as the base. To help improve the density, we performed iterative rounds of refinement and water picking with Phenix^21^ and Coot^22^. However, no significant improvement in the electron density for the incoming dCTP was observed.

We rationalized that the absence of well defined electron density was suggestive of multiple conformations for the dCTP, with only the γ-phosphate ordered and held in place by interaction with positively charged amino acids from the fingers domain (see below). The Polι_1-MeA.dCTP_ ternary complex was refined to 2.0 Å resolution (R_free_ 23.9%; R_cryst_ of 21.6%) and contains Polι residues 26–350, 356–371, 378–397 and 403–414, DNA nucleotides 4–11, 1 Cl^−^ ion and 291 water molecules.

### Overall Arrangement

In both the Polι_1-MeA.dTTP_ and Polι_1-MeA.dCTP_ complexes, a Polι molecule binds to each replicative end of the double-ended template-primer (Fig. 2). The two molecules are related by a crystallographic two-fold axis and thus make identical contacts with the template-primer. Polι has the familiar right-handed architecture with palm (residues 25–37, 99–224), fingers (38–98), and thumb (225–288) domains, and the PAD (polymerase associated domain; residues 298–414) unique to Y-family polymerases^17^,^18^,^23^,^24^. The palm domain forms the floor of the DNA binding cavity and contains the active site residues (Asp34, Asp126 and Glu127) that catalyze the nucleotidyl transfer reaction, whereas the fingers domain drapes over the template 1-MeA in both structures (and over dTTP in the Polι_1-MeA.dTTP_ structure). The thumb domain and the PAD are connected by a long linker that spans the width of the DNA. The thumb skims the minor groove on one side of the DNA duplex whereas the PAD occupies the major groove on the other side. The majority of Polι-DNA interactions are mediated by the PAD, wherein the main chain amides on “outer” β-strands of the PAD β-sheet make a series of hydrogen bonds with the template and primer strands.

### The Polι_1-MeA.dTTP_ ternary complex

The structure reveals how the 1-MeA.dTTP nascent base pair is accommodated in the active site of Polι (Fig. 3a). In previous structures of Polι with template purines (A or G), the steric restraints imposed by the narrow active site of the polymerase are overcome by the template being pushed from the *anti* into the *syn* conformation by the incoming dNTP^17^,^18^,^23^. Rotation of the template 1, *N*^6^-etheneodeoxyadenosine (εdA) to the *syn* conformation has also been observed in the structures of Polι with template εdA and incoming dTTP or dCTP^19^. Template 1-MeA is similarly observed in the *syn* conformation, presenting its Hoogsteen edge for hydrogen bonding with dTTP which remains in the *anti* conformation (Fig. 3b and c). The 1-MeA and dTTP bases are almost coplanar and two putative hydrogen bonds are established between the N6 and N7 atoms of 1-MeA with the O4 and N3 atoms of T (2.8 Å and 3.2 Å respectively). The 1-MeA.T base pair is isomorphic with the A.T and εdA.T base pairs in the structures of Polι_dA.dTTP_ and Polι_εdA.dTTP_, respectively. Superimposition of the Polι_1-MeA.dTTP_ structure with that of Polι_dA.dTTP_ and Polι_εdA.dTTP_ reveals almost perfect overlap between the common N6 and N7 atom of the templating bases and the O4 and N3 atoms of the incoming dTTP.

Incoming dTTP is anchored at one end of the dNTP binding cavity by hydrogen bonding interactions between its γ-phosphate and the side chains of Tyr68 and Arg71 from the fingers domain and Lys214 from the palm domain (Fig. 3a). At the other end, Hoogsteen base pairing with 1-MeA secures the base of dTTP in the binding pocket. The α- and β-phosphates are fixed by interactions with the side chains of Asp126 and Thr65 and with the backbone atoms of Leu35 and Phe38. The dTTP sugar packs against the aromatic ring of Tyr39, and makes a hydrogen bond between its 3′OH and the main chain amide of the Tyr39. A single Mg^2+^ ion (metal B) is coordinated by the triphosphate moiety of dTTP, as well as the active site residues Asp34 and Asp126. Overall, Polι_1-MeA.dTTP_ is well poised for catalysis with a 3′-OH modeled at the primer terminus located ~3.1 Å from the dTTP α-phosphate and aligned more or less linearly with respect to the scissile Pα-O3′ bond (~162°).

### The Polι_1-MeA.dCTP_ ternary complex

Overall, as in the Polι_1-MeA.dTTP_ structure, template 1-MeA is rotated about its glycosidic bond to the *syn* conformation and presents its Hoogsteen edge to the dNTP binding pocket (Fig. 3d and e). However, relative to the template in the incoming dTTP complex, template 1-MeA in Polι_1-MeA.dCTP_ is inclined towards the DNA helical axis by ~10° and protrudes into the minor groove, partially occluding the dNTP binding site (Fig. 3f). As a result of this inclination, the N6 atom of 1-MeA moves by ~1.0 Å into the dNTP binding cavity relative to the incoming dTTP ternary complex.

Another notable difference between the structure of Polι_1-MeA.dCTP_ and Polι_1-MeA.dTTP_ is in the conformation of the catalytic residue Asp126 (Fig. 3f). In the binary complexes of Polι with template purines, Asp126 is involved in hydrogen bonding interactions with solvent molecules that occupy the vacant dNTP binding pocket, as well as a putative hydrogen bond with 3′OH group modeled at the primer terminus^23^. In Polι_1-MeA.dTTP_ (and other ternary structures with purine templates), the side chain of Asp126 undergoes an ~30° rotation to establish new interactions with the backbone carbonyl of Leu35, the metal ion at site B, and with the α-phosphate of incoming dNTP (Fig. 3f)^17^,^18^,^19^,^23^. By contrast, in Polι_1-MeA.dCTP_, Asp126 remains in the same conformation as in the binary structures.

The electron density for incoming dCTP is very weak with only the density for γ-phosphate clearly visible. The “base” of dCTP is poorly defined (Fig. 3e). Attempts to model dCTP using the conformation of dTTP as a guide leads to steric overlap between its N4 amino group and the N6 amino group of 1-MeA. The dCTP C5′ sugar atom and the α-phosphate also clash with the side chain of Asp126. Efforts to relieve these steric clashes by small movements of the base, sugar and phosphate groups of dCTP lead instead to steric clashes with Val64, Thr65 and Tyr39 from the fingers domain or with the template 1-MeA. These steric clashes are aggravated by the narrow active site of Polι^17^,^18^,^19^,^23^,^25^ and protrusion of the template 1-MeA into the dNTP binding pocket by ~1.0 Å. Taken together, the weak electron density for incoming dCTP and the binary like conformation of Asp126 suggest a disordered dCTP with only its γ-phosphate anchored in place while the sugar and base sample a range of conformations.

## Discussion

Alkylating agents modify DNA by adding alkyl groups to both the ring nitrogens and the exocyclic oxygen atoms, generating adducts that have cytotoxic effects^5^,^6^,^8^. 1-MeA is a common adduct generated by the transfer of methyl group to the N1 nitrogen atom of deoxyadenosine. If left unrepaired, 1-MeA presents a strong block to replicative polymerases due to its inability to participate in W-C base pairing. We show here by steady state kinetics that Polι, a Y-family polymerase, is capable of TLS across 1-MeA, and that it incorporates the correct T with an ~100 fold higher efficiency than the incorrect C. We also derive a structural framework for the ability of Polι to accommodate the 1-MeA adduct, and a basis for the selection of correct from incorrect incoming nucleotide.

In all previous ternary structures of Polι with a template purine, the template is in *syn* and dNTP is in *anti* conformation^17^,^18^,^19^,^23^. The Polι active site cleft is narrower than in other polymerases, which effectively pushes the template purine into a *syn* conformation when the incoming dNTP binds. The resulting C1′-C1′ distance across the nascent base pair reduces to <9 Å, favorable for Hoogsteen base pairing. Thus, given the constraints of the Polι active site cleft, it is not surprising that 1-MeA is also pushed in to the *syn* conformation for Hoogsteen base pairing with incoming dTTP (which remains in the *anti* conformation). Importantly, the complex is competent for catalysis with the scissile Pα-O3′ bond of incoming dTTP aligned favorably with respect to a 3′OH modeled at the primer terminus.

By contrast, although the dCTP γ-phosphate occupies the same position as the γ-phosphate of dTTP in the Polι_1-MeA.dTTP_ structure, the rest of the molecule is disordered (Fig. 3e). The ~100 fold lower efficiency of Polι in inserting C relative to T opposite 1-MeA can be rationalized by the inability of dCTP to gain a firm “foothold” opposite 1-MeA. Incoming dCTP does not offer the same hydrogen bonding opportunities opposite 1-MeA as dTTP, and its N4 amino group sterically impinges on the N6 group of 1-MeA. Accordingly, it seems to adopt a range of conformations that is not conducive to catalysis. Also, Asp126 remains in a binary-like conformation and prevents the α-phosphate of dCTP from aligning properly with respect to the primer terminus for catalysis.

Although Polι is inefficient at incorporating the incorrect C (and incorrect A) opposite 1-MeA, it does so more efficiently than opposite undamaged template A (Table 1). We suspect that this is because the methyl group at N1 favors the imino tautomer of 1-MeA^26^. In its imino tautometric form, the N6 imino group of 1-MeA would be in a position to establish a putative N6(1-MeA)…N4(dCTP) or N6(1-MeA)…N6(dATP) hydrogen bond, enabling Polι to incorporate C or A opposite 1-MeA more readily than opposite A.

In conclusion, we present here the first kinetic and structural analysis of the ability of Polι to replicate through the 1-MeA adduct. 1-MeA is highly cytotoxic because a methyl group at N1 atom impairs W-C base pairing and presents a strong block to normal DNA replication. By pushing 1-MeA in to the *syn* conformation, Polι can carry out effective TLS opposite 1-MeA via Hoogsteen base pairing with correct incoming T.

## Methods

### Crystallization

The GST-Polι (residues 1–420) fusion protein was expressed and purified as described previously^27^. A self-complementary 18-mer oligonucleotide was synthesized containing dideoxycytosine at its 3′ end (5′-TCT-1-MeA-GGGTCCTAGG ACCC^dd^-3′, 1-MeA: N1-methyl-deoxyadenosine). Prior to crystallization, the oligonucleotide was annealed with itself to give a “double-ended” template-primer with two replicative ends^18^. For crystallization of the Polι_1-MeA.dTTP_ and Polι_1-MeA.dCTP_ ternary complexes, Polι and DNA were mixed in the ratio of 1:1.2, followed by the addition of dTTP or dCTP and MgCl_2_ to final concentrations of 20 mM and 10 mM respectively. The ternary complexes were crystallized from solutions containing 15–20% PEG 5000 MME and 0.2 –0.4 M (NH_4_)_2_SO_4_ in 0.1 M MES buffer (pH = 6.0). Crystals belong to space group P6_5_22 with cell dimensions of a = 98.0 Å, b = 98.0 Å, c = 202.5/202.2 Å and α = β = 90°, γ = 120°. For data collection, the crystals were cryoprotected by soaks for 5 minutes in mother liquor solution containing 5%, 10%, 15% and 20% and 25% glycerol, respectively, and then flash frozen in liquid nitrogen.

### Structure Determination and Refinement

X-ray data on cryocooled crystals were measured at Brookhaven National Laboratory (BNL beamline X-25) and Advanced Photon Source (APS, beamline 24-ID-E). Data sets were indexed and integrated using HKL2000^28^. The Polι_1-MeA.dTTP_ and Polι_1-MeA.dCTP_ structures were solved by molecular replacement (MR), using the Polι_A.dTTP_ complex as a search model (2FLL, with template and incoming dNTP omitted). The first round of refinement and map calculation was carried out without the template and the incoming nucleotide. Initial electron density map showed unambiguous density for the template 1-MeA in both the structures, which was then included in the model for subsequent refinement. Iterative rounds of refinement and water picking were performed with Phenix^21^ and model building with program Coot^22^. All models have good stereochemistry, as shown by MolProbity^29^,^30^ with >99% of the residues in the most favored regions of the Ramachandran plot and 0.8% in the disallowed regions. Figures were prepared using PyMol^31^.

### DNA Polymerase Assay

DNA substrates consisted of a radiolabeled oligonucleotide primer annealed to a 75nt oligonucleotide DNA template by heating a mixture of primer/template at a 1:1.5 molar ratio to 95 °C and allowing it to cool to room temperature for several hours. The template 75-mer oligonucleotide contained the sequence 5′AGC AAG TCA CCA ATG TCT AAG AGT TCG TAT AAT GCC TAC ACT GGA GTA CCG GAG CAT CGT CGT GAC TGG GAA AAC-3′ and was either undamaged A or harbored a 1-MeA at the underlined position. For steady-state kinetic analyses of nucleotide insertion opposite the undamaged A or 1-MeA, a 44 mer primer 5′ GTT TTC CCA GTC ACG ACG ATG CTC CGG TAC TCC AGT GTA GGC AT-3′ was used annealed to the above mentioned 75 mer templates.

The standard DNA polymerase reaction (5 μl) contained 25 mM Tris·HCl (pH 7.5), 5 mM MgCl_2,_ 1 mM dithiolthreitol, 100 μg/ml BSA, 10% glycerol, and 10 nM DNA substrate, and Polι (0.02–0.2 nM).

### Steady-State Kinetic Analysis

Steady-state kinetic analyses for deoxynucleotide incorporation were performed as described^32^. Polι (0.02–0.2 nM) was incubated with primer:template DNA substrate (10 nM) and increasing concentration of dNTPs for 10 min, at 37 °C. Gel band intensities of the substrate and products of the deoxynucleotide incorporation reactions were quantified by using a PhosphorImager and the IMAGEQUANT software (Molecular Dynamics). The observed rate of deoxynucleotide incorporation, *v*_obs_ was determined by dividing the amount of product formed by the reaction time and protein concentration. The *v*_obs_ was graphed as a function of the deoxynucleotide concentration, and the data were fit to the Michaelis-Menten equation describing a hyperbola: *v*_obs_ = (*k*_cat_[E] × [dNTP])/(*K*_m_ + [dNTP]). From the best fit curve, the apparent *K*_m_ and *k*_cat_ steady-state kinetics parameter were obtained for the incorporation of dNTP by the Polι and the efficiencies of nucleotide incorporation (*k*_cat_/*K*_m_) were determined.

## Additional Information

**How to cite this article**: Jain, R. *et al*. Mechanism of error-free DNA synthesis across N1-methyl-deoxyadenosine by human DNA polymerase-*ι. Sci. Rep.* **7**, 43904; doi: 10.1038/srep43904 (2017).

**Publisher's note:** Springer Nature remains neutral with regard to jurisdictional claims in published maps and institutional affiliations.

## Figures and Tables

**Table 1:** Steady-state kinetic parameters of nucleotide incorporation opposite templates deoxyadenosine (A) and N1-methyl-deoxyadenosine (1-MeA) by human Polι.

Template base	Incoming nucleotide	*k*_cat_ (min^−1^)	*K*_m_ (μM)	*k*_cat_/*K*_m_ (min^−1^ μM^−1^)	^a^Efficiency relative to T opposite same template
A	dTTP	11 ± 0.6	5.3 ± 0.9	2	1
	dATP	0.54 ± 0.02	220 ± 38	0.002	↓10^3^
	dGTP	0.48 ± 0.04	330 ± 70	0.0015	↓7 × 10^4^
	dCTP	ND	ND		—
1-MeA	dTTP	3 ± 0.08	0.28 ± 0.04	10	1
	dATP	5.1 ± 0.28	50 ± 8.1	0.1	↓100
	dGTP	0.69 ± 0.2	750 ± 450	0.0009	↓9 × 10^5^
	dCTP	2.1 ± 0.2	18 ± 7	0.1	↓100

ND- not determined. ^a^Change in efficiency of dNTP incorporation relative to incorporation of T opposite the same template.

**Table 2:** Data Collection and Refinement Statistics.

Data Collection	Polιι_1-MeA.dTTP_	Polι_1-MeA.dCTP_
Space group	P6_5_22	P6_5_22
Cell dimensions (Å)	97.96, 97.96, 202.52	97.92, 97.92, 202.24
Resolution (Å)	2.62	1.96
No. of measured reflections refreflectiponsreflectionsReflections	252003	364863
No. of unique reflections	18059	41925
Completeness (%)^a^	99.7 (99.3)	99.9 (100)
R_sym_ (%)^b^	8.5 (69.5)	7.4 (91.7)
I/σ	27.9 (3.8)	25.2 (2.1)
Refinement Statistics		
Resolution Range	48.96–2.62 (2.71–2.62)	48.96–1.96 (1.99–1.96)
Reflections	18014 (1728)	41847 (1756)
R_cryst_ (%)^c^	20.5 (23.1)	21.6 (27.1)
R_free_ (%)^d^	24.9 (30.0)	23.9 (29.3)
Non-hydrogen atoms		
Protein	2784	2818
DNA	348	332
Incoming dNTP	42	—
Ions	2	1
Water	87	291
B factors (Å^**2**^)		
Protein	59.5	47.6
DNA	57.9	43.1
Incoming dNTP	46.3	—
Ions	68.0	85.5
Water	47.2	45.2
RMS deviations		
Bonds (Å)	0.006	0.005
Angles (°)	0.856	0.697
Ramachandran Plot Quality		
Most favored (%)	96.2	97.0
Generously allowed (%)	3	2.2
Disallowed (%)	0.8	0.8

^a^Values for outermost shells are given in parentheses. ^b^R_sym_ = Σ|I − <I > |/ΣI, where I is the integrated intensity of a given reflection. ^c^R_cryst_ = Σ||*F*_observed_| − |*F*_calculated_||/Σ|*F*_observed_|. ^d^For R_free_ calculations, 7.5% of data excluded from refinement were used.
